# The Role of Antibody Testing and Vaccination in Paediatric Recurrent Acute Otitis Media

**DOI:** 10.7759/cureus.109300

**Published:** 2026-05-20

**Authors:** Karim Awad, Farah Jeffry, Bhaskar Molluru, Huw Jones, Elliot Heward

**Affiliations:** 1 Otolaryngology, University of Lancashire, Preston, GBR; 2 Otolaryngology, Royal Preston Hospital, Lancashire Teaching Hospitals NHS Foundation Trust, Preston, GBR; 3 Otolaryngology, Betsi Cadwaladr University Health Board, St Asaph, GBR

**Keywords:** antibody testing, booster vaccination, otolaryngology, paediatric, recurrent acute otitis media

## Abstract

Introduction: Recurrent acute otitis media (RAOM) is a common condition most frequently affecting children under two years of age. The main causative agent is *Streptococcus pneumoniae*. Pneumococcal vaccination has been shown to be an effective method of RAOM prophylaxis. Currently, pneumococcal antibody testing can be performed to assess immunity levels, followed by booster vaccination if protection is suboptimal. We aimed to explore the role of antibody testing and vaccination in the context of managing paediatric RAOM.

Method: A retrospective study was performed between January and December 2023. A total of 1,004 paediatric referrals (age: ≤ 16 years) to our trust were reviewed. Patients with RAOM who underwent pneumococcal antibody testing were identified. Suboptimal antibody levels were defined as ≥ 6 low serotypes out of 13. This study took place at Royal Preston Hospital, United Kingdom.

Results: Our cohort includes 169 children with RAOM, of which 42 underwent pneumococcal antibody testing with available results. Of these patients, 19 (45.2%) had suboptimal pneumococcal antibody levels; 13 (68.4%) patients received a booster vaccine. A reduction in RAOM episodes was reported in 92.3% (12/13) of vaccinated children at follow-up (≥ 3 months), with improvement in symptoms, including otalgia, fever, and hearing loss. Adequate *Haemophilus influenzae *antibody levels were found in every tested child with RAOM (n=43).

Conclusion: These findings indicate that approximately half of children with RAOM have suboptimal pneumococcal antibody levels, which can be successfully addressed with booster vaccination. Re-testing pneumococcal antibody levels after booster vaccination and performing *H. influenzae *antibody levels are not required. Further work is required to identify if booster vaccinations reduce the frequency or severity of RAOM presentations.

## Introduction

Acute otitis media (AOM) commonly affects children aged ≤ 16 years, with the majority of children experiencing one or more episodes by the age of three [1,2]. Recurrent AOM (RAOM) is defined as having three or more AOM episodes in six months or four or more episodes in the last 12 months [1]. In 95% of cases, RAOM is associated with an upper respiratory infection. Symptoms include otalgia, fever, otorrhea, and hearing loss, which can significantly impact quality of life [1,3].

AOM is most frequently caused by *Streptococcus pneumoniae *(≈35% of cases), followed by *Haemophilus influenzae *(≈20% of cases) [4]. In 5-10% of children, inadequate antibody formation against these pathogens contributes to increased susceptibility to RAOM [3].

In 2012, paediatric AOM presentations were responsible for over 500,000 primary care consultations in the UK [5]. The National Institute for Health and Care Excellence (NICE) recommends referring patients to the ear, nose, and throat (ENT) department if AOM episodes are unexplained, very distressing, or associated with complications [6]. The management of paediatric RAOM includes antibiotic, surgical, and immunological management strategies [2]. Pneumococcal vaccination has been shown to reduce the incidence of RAOM, but the role of antibody testing and booster vaccination remains unclear [3]. Investigating immunological assessment and targeted vaccination could help identify children at risk of recurrent infections and guide individualised management strategies.

This retrospective study focuses on paediatric patients with RAOM referred to a single otorhinolaryngology centre. We aimed to investigate the role of pneumococcal and *H. influenzae *antibody testing and vaccination in managing these patients. We aimed to assess the prevalence of suboptimal antibody levels, response to booster vaccination, and whether routine testing and re-vaccination are warranted in clinical practice.

## Materials and methods

A retrospective cohort study was performed between January 2023 and December 2023 at the Royal Preston Hospital, UK (Local audit registration number: ENT/SE/2024-25/07). Ethical approval was not required according to the Health Research Authority decision tool. Patients were recruited via routine ENT referrals from primary care. A total of 1,004 paediatric ENT (≤ 16 years) referrals were reviewed. Patients were included if they had a diagnosis of RAOM, based on parental history, general practitioner (GP) referral information, and examination if AOM was present at the time of the outpatient appointment. All eligible patients with RAOM were included (n=169 patients), irrespective of coexisting risk factors, such as allergic disease, adenoid hypertrophy, syndromic conditions, immunodeficiency, obstructive sleep apnoea, or craniofacial abnormalities. Referrals unrelated to ear conditions and cases with incomplete data were excluded.

All data were de-identified prior to analysis to ensure patient confidentiality. Demographic data, including age and gender, was collected. Patients who underwent pneumococcal serotype-specific IgG antibody testing and *H. influenzae *type B IgG antibody testing were identified. Suboptimal pneumococcal antibody levels were defined as ≥ 6 low serotypes (< 0.35) out of 13 [7].

Among patients with suboptimal pneumococcal antibody levels, those who received pneumococcal conjugate boosters were identified and assessed to determine whether the intervention led to symptom resolution and achievement of optimal antibody levels on re-testing. Symptom reduction was defined as having fewer episodes than required to diagnose RAOM (< 3 episodes in six months or < 4 episodes in 12 months) [8].

## Results

The cohort included 169 patients with RAOM, comprising 75 males and 94 females, with a median age of three years. Of these, 46 underwent pneumococcal antibody testing, with four excluded due to insufficient samples or unrecorded results. Similarly, 45 patients underwent *H. influenza *antibody testing, with two excluded for the same reasons. Baseline patient characteristics, including age and gender, are summarised in Table 1. No relevant comorbidities were reported.

**Table 1:** Baseline patient characteristics

Characteristic/Outcome	Pneumococcal Testing (n=42)	Haemophilus Testing (n=43)	Total Cohort (n=169)
Median age (years)	2 (range 0-16)	2	3
Sex (M/F)	25/17	22/21	75/94

**Figure 1:**
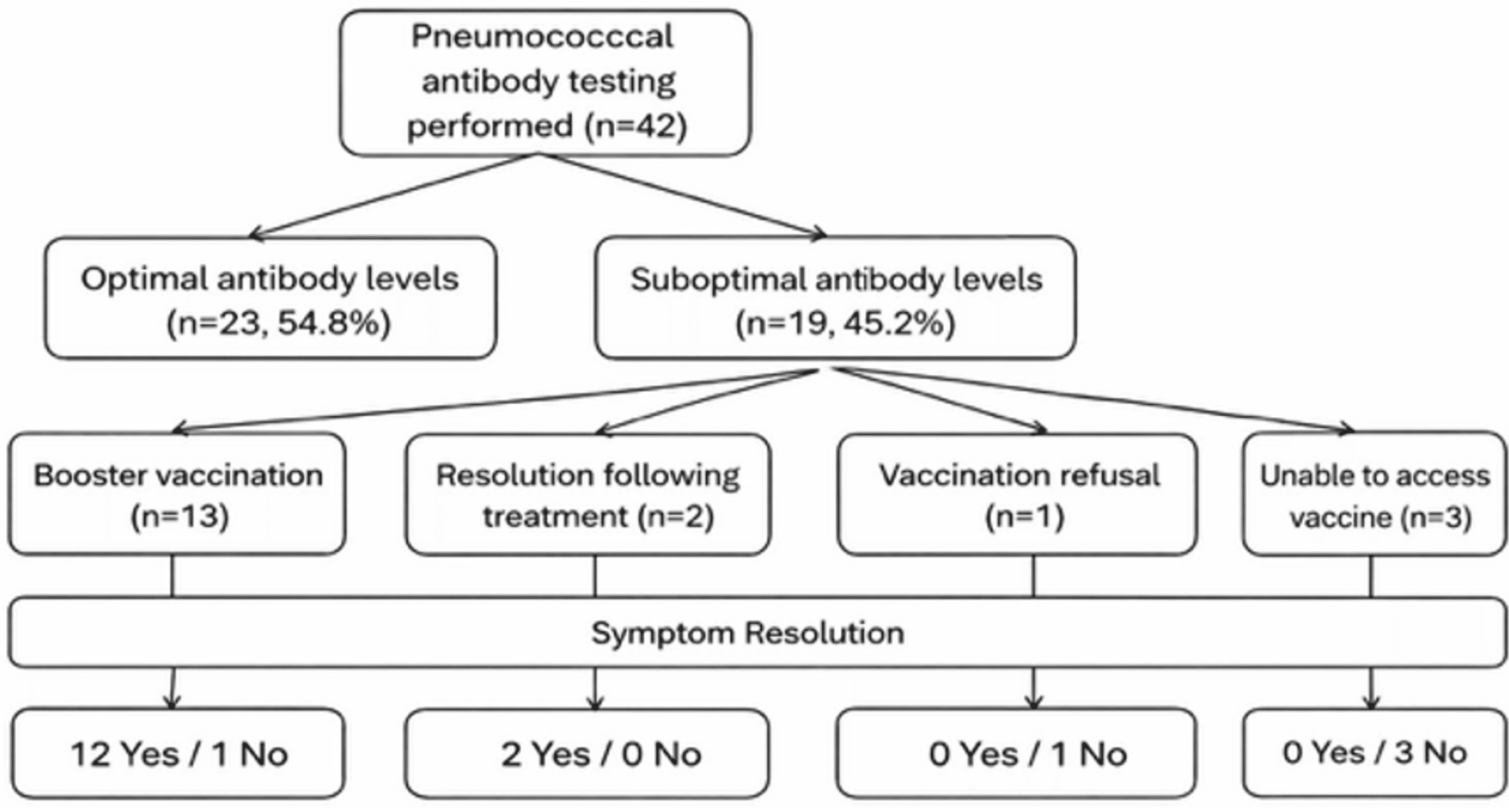
Flow diagram of pneumococcal antibody testing

Among the 42 patients who underwent pneumococcal antibody testing, 25 were male, and 17 were female. The median age of the children tested for pneumococcal antibodies was two years (range: 0-16). Nineteen (n=19/42) patients (45.2%) had suboptimal antibody protection (Figure 1). This subgroup had a median age of two years and consisted of 12 males and 7 females.

Thirteen of these (n=13/19) patients received a booster pneumococcal vaccine. Reasons for not receiving a booster included parental inability to obtain it from primary care (n=3), parental refusal (n=1), symptom improvement after antibiotics (n=1), and symptom improvement following bilateral grommet insertion (n=1). All patients who received the booster vaccine tolerated the intervention without complications. Of the nine patients who were retested post booster, all (100%) achieved optimal antibody levels. At follow-up (≥ 3 months post booster), 92.3% (n=12/13) reported symptom resolution and were discharged. The single patient with ongoing RAOM after achieving optimal immunity was listed for grommets under general anaesthetic. No further ENT referrals related to AOM were made to our trust for these patients (recorded on 27 March 2025). Follow-up for all children was at least three months post-booster vaccination.

In *H. influenza *antibody testing, all 43 children tested had adequate antibody levels, with a median age of two. *H. influenzae *IgG antibody levels were considered adequate if they were ≥ 0.15 µg/mL.

## Discussion

This retrospective series found that, whilst all children with RAOM tested demonstrated adequate *H. influenzae *antibody levels, approximately half (45%) of children with RAOM had suboptimal antibody protection against *S. pneumoniae*. Gender differences in susceptibility to middle ear infections have previously been reported, with males being more susceptible than females [1]. In this study, a slightly higher proportion of male children had suboptimal pneumococcal immunity (12/25, 48%) compared to females (7/17, 41%), which may contribute to this pattern [1]. Booster pneumococcal vaccination appeared effective in restoring antibody levels, with most children showing symptom resolution and optimal antibody levels at follow-up. These results suggest that suboptimal protection against *S. pneumoniae *may play a significant role in the incidence of RAOM.

**Figure 2:**
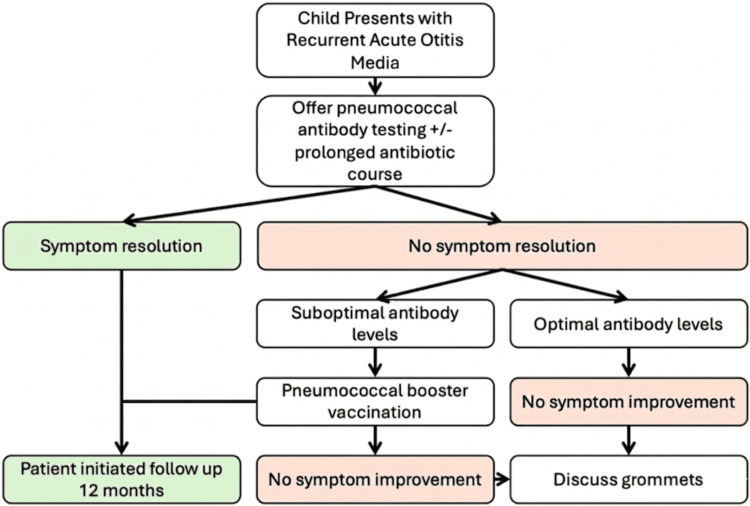
Management algorithm for children with recurrent acute otitis media at our centre

In the UK, children receive pneumococcal vaccinations at 12 weeks and one year of age [9]. Previous research shows that unvaccinated children have a 28% higher risk of RAOM within five years compared to vaccinated children [10]. However, pneumococcal antibody levels are shown to wane over time, potentially leaving children vulnerable to RAOM. *S. pneumoniae *is the main causative agent [11]. This study offers a detailed immunological assessment within a paediatric RAOM cohort, integrating laboratory results of pneumococcal antibody levels pre- and post-booster vaccination, as well as clinical symptom assessment post vaccination. The findings suggest that booster vaccination may restore immunity and reduce AOM frequency, highlighting a potential role for targeted pneumococcal antibody testing and booster vaccination in RAOM management. The results have informed a management algorithm for our centre (Figure 2).

The *H. influenza *vaccine is provided to children at ages 8, 12, and 16 weeks and one year in the UK [12]. Children generate a sustained antibody response, lasting up to six years following vaccination or natural exposure [13]. This likely explains why all children tested had adequate antibody levels. It also suggests that low *H. influenza *immunity is unlikely to be a significant contributor to RAOM, and routine antibody testing for this organism may not be necessary.

Management of paediatric RAOM is heterogeneous, and the role and timing of pneumococcal antibody testing and vaccination remain unclear. At our centre, the cost of a pneumococcal antibody test and vaccine is relatively low (£36.38 and £39.25, respectively) [14]. This appears to be cost-effective compared with repeated antibiotic courses or grommet insertions. Furthermore, booster pneumococcal vaccines carry minimal risk as they are inactivated. The only contra-indication is prior anaphylaxis to the vaccine [9]. The main drawback of this strategy is that venepuncture and vaccination in young children can be challenging and distressing for both the child and family [15].

One of the main strengths of this study is that the findings add to the existing body of knowledge on RAOM and immunological assessment. Furthermore, the absence of loss to follow-up enhances the reliability of the outcome assessment.

On the other hand, this study had several limitations. It is a single-centre study with a relatively small sample size, which may limit generalisability. Furthermore, missing data is a limitation when performing retrospective data collection. Not all children with RAOM had antibody levels tested, which could introduce bias. Similarly, not all children receiving a booster vaccination were re-tested, and the reasons for this were unclear.

Despite these limitations, the findings highlight the need for larger, multi-centre prospective studies to evaluate the effectiveness of pneumococcal boosters in reducing RAOM frequency and severity. Future work should also examine long-term outcomes and cost-effectiveness compared with standard care before considering routine implementation of pneumococcal antibody testing and booster vaccination in RAOM management nationally.

## Conclusions

Our results show that nearly half of children with RAOM have suboptimal pneumococcal antibody levels and that re-vaccination can effectively address this. Among those who received booster vaccination, 92.3% of patients reported no re-occurrence at follow-up. Re-testing pneumococcal antibody levels after booster vaccination and performing *H. influenzae* antibody levels are not required. Importantly, as this study primarily included patients with suboptimal pneumococcal antibody levels, the findings should not be generalised to immunocompetent RAOM populations with adequate baseline immunity. However, further prospective multi-centre studies with larger sample sizes are required to determine whether booster vaccinations reduce the frequency or severity of RAOM compared to other management strategies and to evaluate long-term clinical outcomes and cost-effectiveness.
